# Nutritional Status of Maasai Pastoralists under Change

**DOI:** 10.1007/s10745-015-9749-x

**Published:** 2015-05-19

**Authors:** Kathleen A. Galvin, Tyler A. Beeton, Randall B. Boone, Shauna B. BurnSilver

**Affiliations:** Department of Anthropology, Colorado State University, B-219 Andrew G. Clark Building, Fort Collins, CO 80523-1787 USA; Graduate Degree Program in Ecology, Colorado State University, 238 Natural Resources Building, Fort Collins, CO 80523-1787 USA; Department of Ecosystem Science and Sustainability, A204 NESB - Campus Delivery 1476, Fort Collins, CO 80523-1787 USA; School of Human Evolution and Social Change, Complex Adaptive Systems Initiative, Arizona State University, PO Box 872402, Tempe, AZ 85287-2402 USA

**Keywords:** Human nutrition, Anthropometry, Social-ecological change, Nutrition transition, Maasai pastoralists, Southern Kenya

## Abstract

This study assesses the nutritional status of Maasai pastoralists living in a period of great social, economic and ecological changes in Kajiado County, southern Kenya. Data on weight, height, skinfolds, and circumferences were collected from 534 individuals in the year 2000. The data were used to describe mean differences in human nutrition between ages, sexes, and within and among three Group Ranches. Nutritional data and diet recall data were compared with past studies of Maasai nutrition from 1930 to 2000. Results indicate that nutritional status is poor and has remained so despite numerous changes to the social-ecological system including livelihood diversification, sedentarization, human population growth and decreased access to vegetation heterogeneity. Imbirikani Group Ranch had better access to infrastructure and markets and some measures of nutritional status were better than for individuals in other group ranches. However, nutritional status remains poor despite transitioning to greater market integration.

## Introduction

The food production systems of the peoples of the drylands of East Africa are based on livestock and, rainfall permitting, some cultivation. However, recurrent and extreme weather events and changes in markets, land tenure, population and urban growth have greatly affected these production systems (Galvin 2009; Reid *et al.*^2014^). The 2000 decade saw several severe droughts, in 2000, 2005–6 and in 2009. There was an increased reliance on market involvement and cash transactions—what would be expected of a population experiencing a transition from subsistence production to increasing dependence on market-driven goods and services including purchased foods, clothing, household articles, health care, and veterinary services. Processes of increased subdivision of land and sedentarization have fragmented the landscape making it difficult for herders to move their livestock, the primary management strategy of pastoralists (BurnSilver  *et al*. 2008; Hobbs *et al.*^2008^). As human populations have grown, livestock to human ratios have decreased, spurring the need for livelihood diversification. Settlements are increasingly closer to villages as people seek better access to schools, health care, jobs and water.

This study documents the nutritional status of Maasai pastoralists within its changing social, economic and ecological context. Economic change and its attendant social-ecological effects such as livelihood diversification, new settlement patterns and land fragmentation influence food production patterns. There is an important and complex relationship between the environment, economic status, lifestyle and nutritional status. Accompanying environmental, economic and social changes is often a change in labor and work tasks with an increased reliance on purchased foods. This is accompanied by a shift in diet and a reduction in physical activity, both of which affect people’s nutritional status. This process is termed a nutrition transition (Popkin 2004, 2006). The present study uses anthropometry to assess if a nutrition transition is occurring among Maasai pastoralists.

Anthropometric indices are highly reliable and sensitive indicators of growth and body composition. They are the single most widely used measure of nutritional status because of their precision, replicative nature and the availability of accurate standards for comparison. Therefore, relatively simple measures of weight and height, with information on age and sex can yield reliable information on nutritional status (Frisancho 2011). For example, height compared to age is a good indicator of the long-term nutritional status of a child, whereas weight compared to height is thought to be a good assessment of the current health status of a child. Triceps skinfolds is a simple measure of the body’s fat stores whereas upper arm circumference combined with triceps skinfold provides an indicator of protein stocks.

## Conceptual Framework

We utilize two frameworks to understand the nutritional state of Maasai pastoralists. They include the nutrition transition model for understanding the effects of change on nutrition and a social-ecological systems framework for conceptualizing the important components of change and how they interact to affect nutritional status.

A nutrition transitions framework examines diet and nutrition within the context of shifts in land tenure and land use changes, human population growth, livelihood diversification, rural-to-urban migration, sedentarization and climate change (*e.g*., Dufour and Piperata 2004; Olszowy *et al*. 2012; Piperata *et al.*2011b; Popkin 1993, 2004). The nutrition transition framework encapsulates two interrelated phenomena, the demographic transition, where there is a shift from high fertility and high mortality to low fertility and aging populations, and an epidemiological transition, where systems change from populations characterized by high rates of infectious disease to a system characterized by increases in non-communicable and degenerative diseases (Popkin 1993; Popkin 2004). Obviously, these scenarios are two ends of a spectrum and there are undoubtedly situations in which populations are experiencing any degree of these transitions. Many of the case studies that test the nutrition transition do so in the context of highly industrialized urban environments (Popkin 1993, 2004), wherein links have been made to changes in physical activity and diet composition to increased rates of obesity, Type II diabetes, hypertension and cardiovascular disease. However, there are also several studies that address nutritional changes that are occurring in traditionally rural communities that may not be experiencing truly urbanized or industrialized circumstances, but whose members’ nutritional status is influenced by social and economic changes (*e.g*., Dufour and Piperata 2004; Olszowy *et al*. 2012; Piperata *et al*. 2011b). Studies focused on market integration address market impact on nutritional status, with increased market integration associated with increased rates of overweight and obesity, whereby reductions in physical activity levels are manifested in reduction in muscle mass and an increase in arm fatness (Piperata *et al.*2011a, b).

Abandoning traditional lifestyles or greatly altering them while increasing access to markets results in an increased dependency on purchased foods (Ianniotti and Lesorogol 2014). Concomitant with lifestyle changes, nutrient dense diets may decrease while consumption of carbohydrate-rich, fatty, and low-fiber foods increases, as a result from a shift away from local food production to a greater reliance on purchased goods. It is suggested that in some cases as people move closer to town they become worse off (Dufour and Piperata 2004; Fratkin *et al*. 1999). They are typically in a poorer social-economic situation in urban and peri-urban environments and may in fact share a double burden of disease, meaning that these individuals are susceptible to both infectious disease and non-communicable diseases. Their nutritional status is thought to be lower than those who have not experienced the nutritional transition and those who are long-term urban dwellers (Dufour and Piperata 2004).

We also use a social-ecological framework which recognizes that human and ecological well-being is tightly linked, especially in systems where people are reliant directly on the environment for their livelihoods (Myers and Patz 2009). Grace *et al*. (2012) found that variability in climate across Kenya was correlated to malnutrition. Further, diversifying incomes and loss of landscape complexity were linked to lower nutritional status in Brazil (Adams *et al.*^2013^). We acknowledge that the link between social-ecological changes and human health are complex. We cannot directly link human health to specific social or environmental changes in a direct cause and effect relationship, but we describe a series of ecological and social changes and document nutritional status through time. Land use and livestock management strategies provide services (food, forage and water) that sustain human health, but there is little research to date linking them at the household level (Myers and Patz 2009; Tallis *et al.*^2013^). We try to do this indirectly.

Nutritional research was conducted in May and June 2000 as part of a broader study to assess livelihood strategies and human wellbeing of Maasai pastoralists under change (see BurnSilver 2009; BurnSilver *et al*. 2008), under the auspices of the Livestock-Climate Change Collaborative Research Support Program (Boone *et al*. 2002; Galvin *et al*. 2002; BurnSilver *et al*. 2003; Thornton *et al*. 2003; Galvin *et al*. 2004). The many changes in Kajiado County, such as an increased reliance on cash, greater market involvement, and division of communal lands into group ranches (GR) and their further subdivision into individually owned parcels, have affected the economic strategies, lifestyles and living conditions of the Maasai people similar to what would be expected of a population experiencing a nutrition transition.

We use nutritional status derived from anthropometric indices and diet recall data to test hypotheses concerning nutritional status in the context of a pastoral system transitioning through time. In order to make comparisons across time, we examine our data qualitatively in light of previously published reports of nutritional status of Maasai in Kenya and Tanzania from the 1930’s up to 2000 (Orr and Glick 1931; Nestel 1986; McCabe *et al*. 1989, 1992; our study). We address the following hypotheses:

1. Nutritional status is poor for all age/sex groups.
2. Nutritional status in un-subdivided and subdivided GRs is similar.
3. Nutritional status remains relatively stable over time (1930–2000) despite significant, diverging social, economic and environmental changes across Maasailand.
4. Pastoral diets vary between GRs. Those GRs closer to towns have more diverse diets.

## Study Area

### Kajiado County under change: Populations, livelihood diversification, land use/tenure changes, market changes, climate, ecology

Kajiado County is an area of mixed grassland-shrub on volcanic soils. Precipitation ranges between 400 to 800 mm and occurs in a bimodal pattern over two rainy seasons and two dry seasons. This semi-arid to arid region has a south to north rainfall gradient with recurring droughts, and rainfall patterns even in good years are spatially and temporally variable. Once governed by communal land tenure and use, Kajiado County (when this study was conducted it was a district but became a county under the new Kenyan constitution of 2010) was adjudicated into GRs beginning in the 1960s. This effort was applied to communal lands nationwide, led by the Kenyan government and supported by the World Bank under the assumption that private property would be a more rational and productive basis to support the transition from subsistence pastoralism to a system of intensive livestock production (Oxby 1982, Nkedianye 2009). What had been divided into eight sections historically recognized by the Maasai was divided into about 54 GRs and groups of registered pastoral households were granted leasehold tenure under a framework of group freehold tenure (Kimani and Pickard 1998). Maasai pastoralists conversely viewed the GR scheme as a means to maintain control over their land (Galaty 1980). Early in the creation of GRs, influential people (Maasai and some non-Maasai) acquired title to individual parcels. Ownership of individual parcels was condoned by the government in the 1980s, and subdivision commenced. The initial result was fragmentation of the landscape into GRs interspersed with private parcels. Even as the process of GR formation in Kajiado continued through the 1980s, the process of subdivision of GR land into private parcels began, particularly in the wetter (northern) areas of the district. GRs were envisioned by policymakers as an intermediate step between communal land tenure and eventual privatization of land down to the level of household parcels, predicated on the idea that limiting pastoral livestock mobility and increased provision of veterinary and market outlets would lead to a decrease in livestock stocking rates and more market oriented livestock production strategies. Much has been written regarding the failure of this larger strategy to affect intended changes (Galaty 1992), however the GR system did gradually increase infrastructure availability for pastoral households, for example leading to the establishment of permanent water points, veterinary facilities and livestock markets. While subdivision of GRs has continued, the arid regions of Kajiado have largely remained organized as GRs, although areas with access to irrigated water or higher altitude lands have been subdivided informally into agricultural plots and distributed to GR members.

A second, concurrent process of sedentarization has been ongoing even within those GRs as yet unsubdivided, as pastoral households have settled permanently around permanent water, infrastructure and agricultural zones (*e.g*., Namelok swamp in southern Kajiado). Thus the current pastoral landscape in Kajiado is a mosaic of sedentary agropastoralism in high potential agricultural areas, and more extensive pastoralism in unsubdivided, drier and more infrastructure poor regions (BurnSilver and Mwangi 2007). This describes the situation in the three study GRs of Imbirikani, Olgulului-Lolarashi and Eselengei when nutritional data were collected in 2000.

Increasing frequency of droughts and changes in seasonality along with increased settlement near infrastructure has increased pressures to diversify livelihoods. It has also caused production strategies to become more individualized, even as livestock remains at the core of household livelihoods across the region (BurnSilver *et al*. 2008). That said, Maasai have become less reliant on strict livestock-based foods (*e.g*., milk and meat) as they diversify their livelihoods into agriculture, wage labor and business (BurnSilver 2009). In the classic sense, members of Maasai households increasingly engage in multiple activities (Homewood *et al*. 2009). However, diversification pathways also have a mobility component. Households may diversify spatially, so that large, extended households take advantage of opportunities to carry out irrigated or highland rainfed agriculture in southern Amboseli, but simultaneously keep their livestock in areas with better grazing. BurnSilver 2009 found that 31 households from a total sample of 184 (17 %) spatially diversified their livelihood activities in this way, with associated creative divisions of household labor between locations. Heads of these households often traveled between settlements to check on agricultural and livestock activities. Rural to urban seasonal migration by members of Maasai households also occurs (May and Ole Ikayo 2007), whereby migrants send or bring money back to their families periodically. Table 1 summarizes the changes that have occurred in Kajiado County over the last 40 years.

**Table 1:** Social-ecological changes in Kajiado County

Change	Past	Present	Reference
Land tenure	Communal	Mosaic of group ranches (Group Leasehold Title) and individual parcels (Private Property)	Reid *et al*. 2008; Kimani and Pickard 1998
Economic activity	Livestock based subsistence consumption	Diversified; agropastoralism to extensive pastoralism with market engagement	BurnSilver *et al*. 2008
Livestock production	Extensive; seasonal transhumance	A mosaic of landuse: sedentary agropastoralism--> Extensive seasonal transhumant pastoralism	BurnSilver *et al*. 2008
Mobility type Frequency of movement	Open Often-entire HHs moved	Limited in subdivided areas and in sedentary areas (eg. swamps and highland agricultural areas). Livestock herds only move seasonally with young men or hired herders	BurnSilver *et al*. 2008; BurnSilver and Mwangi 2007
Sedentarization	Low (few houses)	High in agricultural areas. All HHs have permanent homes regardless of mobility patterns	Worden 2007
Human population density	Low	High	BurnSilver 2009
Biodiversity	High	Some species in decline	BurnSilver 2009; Reid *et al*. 2008
Vegetation heterogeneity access	High access	Low access	BurnSilver *et al*. 2008
Rainfall variability		Increasing	Ogutu *et al*. 2007
Drought severity frequency	Lower	HIgher	Ogutu *et al*. 2007

**Fig. 1:**
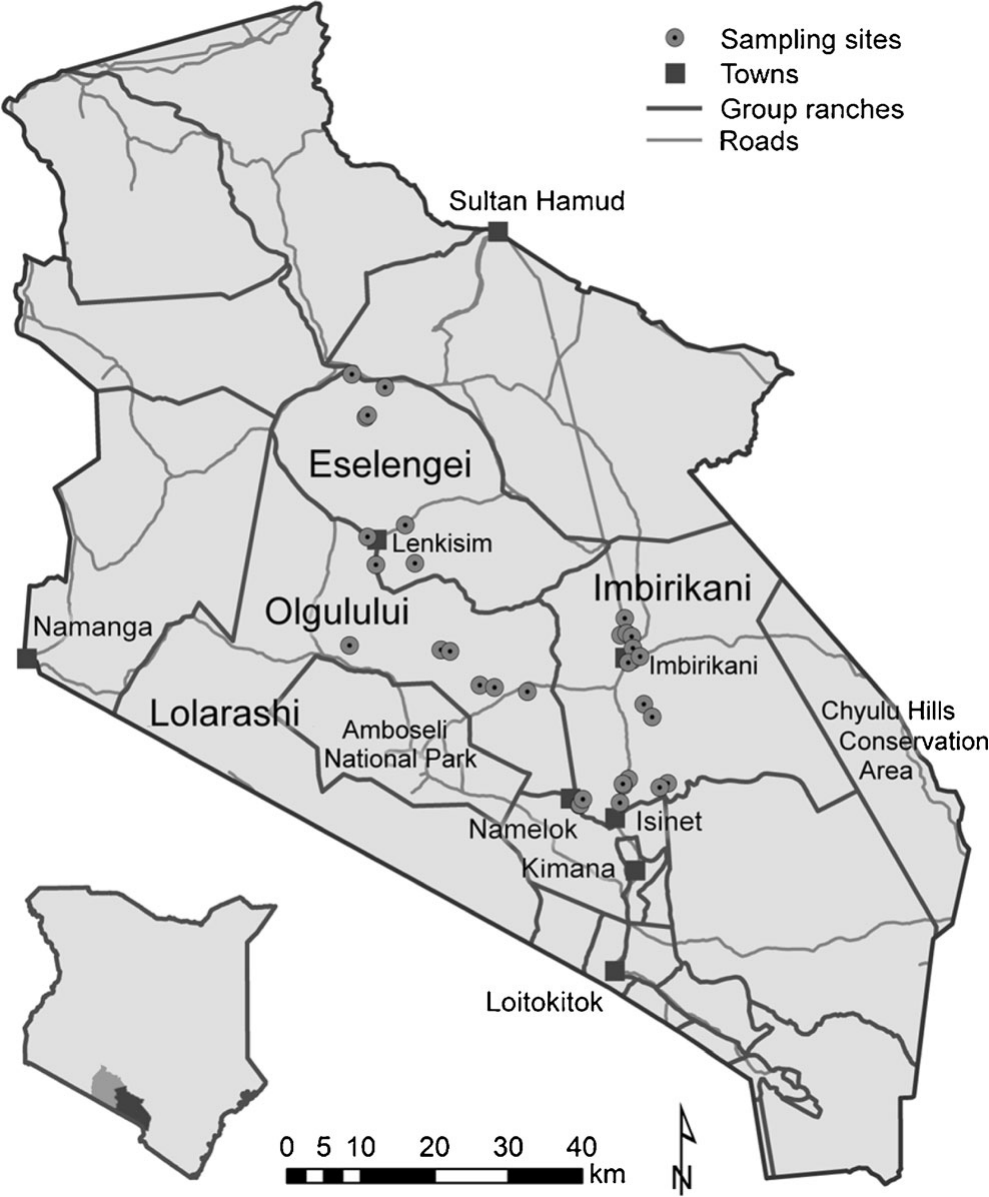
Study area and associated group ranches

Figure 1 is a map of the three study sites. Each dot on the map represents a GPS point of a group of households who participated in the anthropometric study. Table 2 compares the GRs in terms of area, population size, distance to town, land tenure, land use, infrastructure access, agroecological potential, TLUs per person (adult unit), income, mobility, and distance traveled during seasons.

**Table 2:** Group ranch characteristics for Imbirkani, Eselengei and Olgulului/Lorashi group ranches

Group ranch characteristics	Imbirikani_a_	Eselengei_a_	Olgulului/Lorashi^a^
Area (km^2^)	1361	797	1566
Average distance to nearest village (km)	2.4	12.9	17.2
Land tenure	Communal	Communal	Communal
Land use	Extensive-sedentary	Extensive	Extensive
Infrastructure access	Medium-High	Low-Medium	Very low
Mean TLU per AU_b,c_	5.55	6.55	8.7
Gross livestock income ($)	1177	959.5	1415
% HH with agricultural income	64	20	31
% HH with off-land income	49	63.5	52
Mean HH off-land income ($)	661.5	675.5	297
% HH with wildlife-based income	12.5	6.5	10
Mean HH wildlife-based income ($)	1,258	642	191
% HH mobile	78	95.85	95.7
Mean number of moves per year	3.25	2.95	1.9
Daily mean distance traveled (wet)	8.2	6.55	9.9
Daily mean distance traveled (dry)	10.4	10.2	8.7

Group ranch characteristics adapted from BurnSilver *et al*. 2008; BurnSilver 2009

^a^Average of study areas in GR: Imbirikani = N. & S. Imbirikani; Eselengei = Eselengei & Lenkisim; Olgulului/Lorashi = Emeshenani (Burnsilver *et al*. 2008; BurnSilver 2009)

^b^TLU (Tropical Livestock Unit): exchange ratio as function of body and metabolic weight so that different species of varying sizes may be compared using standard units (1 TLU = 250 kg Cattle)

^c^AU (Adult Unit): standard reference adult, based on food or metabolic requirements. Adult male = 1 AU; adult female = 0.9; M/F 10–14 years = 0.9; M/F 5–9 years = 0.6; MF 2–4 = 0.52 (Homewood and Rogers 1991)

## Methods

### Data Collection and Analysis

In May-June 2000, we collected anthropometric measurements from 1000 individuals ranging from infants to 66 years of age, and included household data from the three GRs described above, Imbirikani, Eselengei, and Olgulului/Lorashi, in addition to nutritional data collected from the Imbirikani Dispensary and primary school. The aim of the present study was to examine nutritional status and change at the household level. Therefore, the present analysis discusses results from the three GRs in Kajiado County, Kenya only. A total of 534 people from 46 households are included in this sample. Table 3 shows the age and sex distribution of the sample, indicating more females than males. As is typical of pastoral populations, boys and men are often away from the homestead herding livestock and other tasks (*e.g*., taking livestock to markets, engaging in off-land business, etc.). All data collection methods were reviewed and approved by the Human Subjects Institutional Review Board (IRB) at Colorado State University.

**Table 3:** Age and sex distribution of study sample participants

Age category	♂	♀	Total
Infants (0–1.9)	31	30	61
Children (2–6.9)	56	64	120
Juveniles (♂ 7–11.9; ♀ 7–10.9)	17	33	50
Adolescents (♂ 12–17.9; ♀ 11–17.9)	19	56	75
Adults (18+)	96	132	228
Total	219	315	534

#### Age and Anthropometry

Anthropometric measurements were recorded for each present member of the household; the ages of all participants were obtained by individual or family recall. Individuals were categorized according to biologically meaningful age groups: a) infants (0–1.9); b) children (2–6.9); c) juveniles (males—7–11.9, females—7–10.9); d) adolescents (males—12–17.9, females—11–17.9); and e) adults (18+) (Table 3). Measurements were obtained via standardized procedures from Lohmann *et al*. (1988). We used a home-made measuring board to obtain infant length  to the nearest 1 mm, while a spring balance was used to record infant weights to the nearest 10 g.  In a few instances, the mother was weighed holding the infant and the mother’s weight subtracted, which may have introduced some unreliability to the data by not being exactly accurate. For individuals over the age of two, we recorded heights to the nearest 1 mm, weights to the nearest 100 g, triceps skin fold (TSF) to the nearest 0.5 mm using a Lange skinfold caliper, and upper-arm circumference (UAC) to the nearest 1mm using a flexible graduated tape measure. All measurements were taken by KG.

Additional anthropometric indices were derived from the metrics listed above. Body mass index (BMI) was calculated using the comprehensive database from Frisancho (2011) as follows: BMI = (weight(kg)/(height(m))^2^. Upper-arm muscle area was calculated as: UMA (cm^2^) = [UAC – (3.1416 * TSF)]^2^/12.57 (Piperata *et al*. 2011b).

#### Human Nutrition

We used the anthropometric data to describe mean differences in human nutrition between ages, sexes, and also within and between GRs when applicable. Z-scores were derived from Frisancho (2011), a comprehensive database which includes standardized anthropometric scores from the National Health and Nutrition Examination Surveys (NHANES III), World Health Organization (WHO 2006), and Center for Disease Control (Kuczmarski *et al*. 2002). Weight-for-height (WHZ), height-for-age (HAZ), and body mass index (BMIZ) z-scores were calculated for all individuals under the age of 5 in accordance with WHO reference standards (WHO 2006). The NHANES III was used to obtain HAZ and BMIZ scores for individuals greater than 5 years old, and triceps skin fold (ZTSF), and upper-arm muscle area (ZUMA) for all individuals greater than 2 years of age. According to Frisancho (2011), the NHANES III reference standards are appropriate for individuals who are over 2 years of age if the data were collected to the nearest year, which is the case for individuals older than 2 in the present study, or for individuals older than 20 years of age. Hence, we believe that the procedures carried out here comply with standardized reference materials.

We used several z-scores as proxies of short-term and long-term nutritional status following Piperata *et al*. (2011b). HAZ was used to assess long-term nutritional status for all individuals; a z-score less than or equal to −2.0 indicated the prevalence of stunting (WHO 2006). WHZ was used to asess short-term nutritional status in infants, and wasting was inferred from a z-score of less than or equal to -2.0 (WHO 2006).  BMIZ was used to assess short-term nutritional status in sub-adults (between 2 and 18 years of age), where individuals below the 5th percentile were considered underweight, those falling between the 85th and 95th percentile were considered overweight, and those above the 95th percentile were considered obese (de Onis *et al*. 2007; Must *et al*. 1991). Adult BMI categories were used to assess short-term nutritional status; less than 18.5 (underweight), 18.5–24.9 (normal), 25–29 (overweight), and > 30 (obese) (WHO 1995). For individuals greater than 2 years of age, we assessed fat stores and protein reserves using ZTSF and ZUMA, respectively. The prevalence of malnourishment was indicated by a ZUMA score less than or equal to -2.0 (Frisancho 2011).

We first report differences in z-scores between the sexes and age categories with all ranches combined. One-way analysis of variance (ANOVA) and the appropriate corresponding post-hoc procedure (Scheffe or Dunnet’s C for samples that did not conform to the homogeneity of variance assumption) were used to identify differences between age categories among male and female participants, while significant gender differences for each age category were interpreted using an independent sample t-tests. Significant gender effects in stunting, wasting/underweight, overweight/obese, and malnutrition in sub-adults, in addition to adult BMI categories were tested using the chi-square test.

Our adult sub-sample was large enough to identify significant differences with regard to gender within individual GRs as well as the differences between ranches, while controlling for gender. A similar analytical approach to that explained above was applied to the adult sub-sample to explore differences in adults within GRs and between them. The analytical procedures presented herein, are adapted from Piperata *et al.* (2011b). Table 4 shows the number of male and female adult individuals from each GR analyzed.

**Table 4:** Adult only sample by sex and group ranch

	Imbirikani	Eselengei	Olgulului/Lorashi	Total
♂	47	33	16	96
♀	50	55	25	130
Total	97	88	41	226

#### Maasai Nutritional Status: 1930–2000

We present results from previous studies on Maasai in Tanzania (McCabe *et al*. 1989; Orr and Gilks 1931) and Kenya (Nestel 1986) over the course of the last 85 years as a means to compare whether substantial changes in nutritional status have occurred. The data consist of published aggregate data only, and therefore preclude testing for significant differences using inferential statistical procedures. However, we can compare basic descriptive statistics between individual data sets with reference to CDC (Kuczmarski *et al*. 2002) and WHO (2006) standards.

#### Diet Recall

Twenty-four hour diet recall data were collected from a subsample of households (*n* = 20). Eighty-six interviews were conducted, where respondents were asked the types of foods consumed the day before. We know that urbanization and peri-urbanization can lead to a dietary transition. For agriculture, the nutritional transition is normally a move from a locally-produced diet to a modern diet high in refined carbohydrates (Lourenco *et al*. 2008; Santos *et al*. 2013). For pastoralists however, the transition may be from a diet high in protein and low in energy to a higher carbohydrate diet but lower protein intake. We do not have the diet intake data to test this however. Instead, we compare diets by GR to distance to market centers. We test the simple assumption that pastoralists who live closer to towns have a more diverse diet.

## Results

### Human Nutrition

Table 5 reports the mean z-scores for our sample population and indicates significant differences between the sexes and between age groups. We only report those differences that were significant at the *P* < 0.05 or below.

**Table 5:** Mean comparison of height-for-age (HAZ), weight-for-height (WHZ), body-mass index (BMIZ), under arm muscle area (ZUMA), and tricep skin fold (ZTSF) z-scores by age and sex across all group ranches

	Sex	Infants	Children	Juveniles	Adolescents	Adults
HAZ	♂^a^	−1.31	−1.57	−0.92	−1.58	−0.25
	♀^b^	−1.43	−1.21	−1.26	−0.92	−0.19
	*P*-Value	NS	NS	NS	<0.01	NS
WHZ/BMIZ	♂	−0.04	−1.42	−1.88	−1.81	−1.54
	♀^c^	−0.06	−1.62	−1.79	−1.37	−1.27
	*P*-value	NS	NS	NS	<0.01	<0.01
ZUMA	♂^d^	–	−2.04	−2.30	−2.53	−2.04
	♀^e^	–	−1.46	−1.86	−1.31	−0.91
	*P*-value		0.03	0.02	<0.001	<0.001
ZTSF	♂^f^	–	−0.29	−1.25	−0.75	−0.43
	♀	–	−0.59	−0.95	−0.87	−0.83
	*P*-Value		NS	NS	NS	<0.01

*P*-Values shows significant differences between male and females

^a^Male adults have significantly higher HAZ than all male groups except for Juveniles (*F* = 22.07, *P* < 0.01)

^b^Female adults have significantly higher HAZ than all other female groups (*F* = 20.4, *P* < 0.01)

^c^Juvenile females have significantly lower BMIZ than adolescent and adult females (*F* = 5.22, *P* < 0.01)

^d^Male adults have significantly higher ZUMA than male adolescents (*F* = 3.02, *P* < 0.05)

^e^Female adults have significantly higher ZUMA than any other group; Female adolescents have significantly higher ZUMA than female juveniles (*F* = 19.34, *P* < 0.01)

^f^Male children have significantly higher ZTSF than male juveniles; male adults have significantly higher ZTSF than male juveniles (*F* = 3.99, *P* < 0.01)

#### Differences Between Age Groups

Mean HAZ differed between age groups for both males and females. Males satisfy the test for homogeneity of variance, therefore the ANOVA P-value can be considered accurate. Although the female subgroup does not satisfy the assumption of homogenous variances, the Brown-Forsythe and Welch test for equality of means offer confidence in the conclusion that there is a significant difference in female HAZ between age groups. The Scheffe post-hoc tests suggest that male adults have higher HAZ than all male groups except for the juvenile cohort, while female adults exhibit significantly higher HAZ than all other female groups. Adolescent and adult females have higher BMIZ scores when compared to juvenile females (*F* = 5.22; *P* < 0.05). Additionally, ZUMA differed between age groups in both male and female participants. Male adults have higher ZUMA than male adolescents. Female adults have higher ZUMA scores than any other age group and female adolescents have higher ZUMA when compared to female juveniles. With regards to ZTSF male children and adults have higher ZTSF when compared to male juveniles.

#### Differences Between Sexes

Independent samples t-tests identified the differences between sexes within particular age groups. Female adolescents have higher HAZ when compared to male adolescents in our sample. Additionally, BMIZ scores for female adolescents and adults were higher than male adolescents and adults. Female ZUMA scores were higher when compared to male ZUMA scores for each age group. Adult males exhibit higher ZTSF scores when compared to adult females.

The chi-square test identified significant gender differences and is indicated by bold type in Table 6. There is a gender effect in adolescents with regard to BMI (wasting/underweight) as more males are underweight when compared to females. The prevalence of malnourishment (ZUMA < −2.0) is different between the sexes for all groups besides the juvenile subgroup; males have a higher frequency of malnourishment than do females in the sample.

**Table 6:** Proportion of individuals by age and sex who are stunted, wasted/underweight, overweight, obese, and/or malnourished

Age Group	Infants		Children		Juveniles		Adolescents		Adults	
Sex	♂	♀	♂	♀	♂	♀	♂	♀	♂	♀
Stunted	28.6 %	44.0 %	27.3 %	26.9 %	21.4 %	24.1 %	*27.8* %	*7.5* %	0.0 %	2.3 %
Wasted/Underweight	8.0 %	6.9 %	45.2 %	51.1 %	70.6 %	71.0 %	***68.4*** %	***32.1*** %	51.0 %	59.0 %
Overweight/Obese	–	–	2.4 %	2.2 %	0.0 %	0.0 %	0.0 %	0.0 %	4.1 %	4.6 %
Malnourished	–	–	***75.0*** %	***31.8*** %	71.4 %	48.3 %	***91.7*** %	***12.7*** %	***52.0*** %	***1.5*** %

Boldface type indicates significant gender effect (*p* < 0.05)

#### Comparing Adult Subset Across GRs

Here we describe differences in adult mean z-scores, and gender differences in BMI categories and the prevalence of malnourishment both within and between individual GRs. Table 7 summarizes z-scores differences and Table 8 illustrates results from gender differences using the chi-square test. We describe only those differences that are statistically significant below.

**Table 7:** Mean comparison of adult height-for-age (HAZ), body-mass index (BMIZ), under-arm muscle area (ZUMA), and triceps skin fold (ZTSF) z-scores by group ranch and sex

Group ranch		♂	♀	*P*-Value^a^
HAZ	Imbirikani	−0.21	−0.11	NS
	Eselengei	−0.39	−0.19	NS
	Olugulului/Lolorashi	−0.06	−0.34	NS
	*P*-Value^b^	NS	NS	
BMIZ	Imbirikani	−1.41	−0.99	<0.05
	Eselengei	−1.62	−1.46	NS
	Olugulului/Lolorashi	−1.77	−1.43	NS
	*P*-Value	NS	<0.01^c^	
ZUMA	Imbirikani	−1.86	−0.83	<0.01
	Eselengei	−2.26	−1.04	<0.01
	Olugulului/Lolorashi	−2.21	−0.81	<0.01
	*P*-Value	<0.05^d^	NS	
ZTSF	Imbirikani	−0.39	−0.81	<0.05
	Eselengei	−0.37	−0.75	NS
	Olugulului/Lolorashi	−0.70	−1.07	NS
	*P*-Value	NS	NS	

^a^Independent sample *t*-test shows significant difference between sexes

^b^ANOVA p-value shows significant differences between group ranches; see special notes below

^c^Females in Imbirikani have significantly higher BMIZ than females in Eselengei (*F* = 6.17, *P* < 0.01)

^d^Males in Imbirikani have significantly higher ZUMA than males in Eselengei ((*F* = 4.04, *P* < 0.05)

**Table 8:** Proportion of individuals who are characterized as underweight, overweight/obese, and malnourished by group ranch and sex

Group ranch	Sex	Underweight	Overweight/Obese	Malnourished
Imbirkani	♂	38.3 %	2.1 %	**41.5 %**
	♀	43.1 %	9.8 %	**1.9 %**
Eselengei	♂	57.6 %	9.1 %	**65.4** **%**
	♀	70.4 %	1.9 %	**1.8** **%**
Olugulului/Lolorashi	♂	75.0 %	0.0 %	**62.5** **%**
	♀	68.0 %	0.0 %	**0.0** **%**

Boldface type indicates significant gender differences within group ranches (*P* < 0.001)

#### Differences Between Male and Female Adults Within GRs

There are several differences that emerge when looking at average z-scores between male and females by GR. Females have higher BMIZ scores when compared to males in Imbirikani (Table 7). Second, females have higher ZUMA scores when compared to males in all three GRs (Table 7), though the limited number of male individuals with reported ZTSF in Olgulului/Lorashi GR (*n* = 8) reduces our confidence that the statistical results for that particular GR are accurate. Third, ZTSF is higher in males in Imbirikani GR (Table 7). The prevalence of malnourishment is gender biased; for all GRs, a higher percentage of males were classified as malnourished when compared to females (Table 8).

#### Differences Between GRs

Two significant differences emerged when controlling for gender. First, females in Imbirikani GR have higher BMIZ than females in Eselengei (Table 7). Second, males in Imbirikani GR have higher ZUMA than males in Eselengei GR (Table 7). There is no statistical difference in the prevalence of underweight and malnourishment between the group ranches, however it is interesting to note that Imbirikani has the fewest individuals who were classified as underweight and malnourished when compared to the other GRs (Table 8).

#### Maasai Nutritional Status Over Time: 1930–2000

**Fig. 2:**
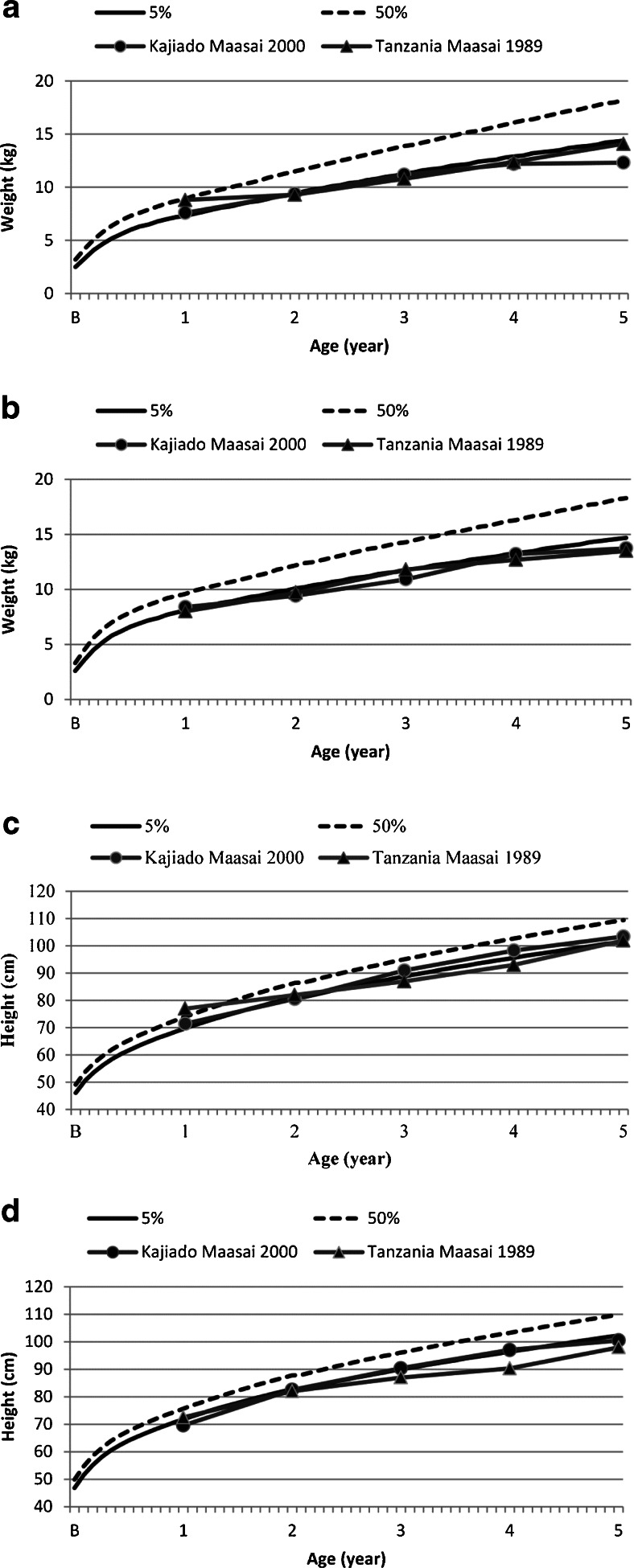
Mean comparison of boys and girls height for age and weight for age in reference to 5th and 50th percentile standards (Kuczmarski *et al.* 2002). We compared our study sample to a Maasai sample from Tanzania (McCabe *et al*. 1989, 1992): **a**) girls weight for age; **b**) boys weight for age; **c**) girls height for age; **d**) boys height for age

In this section, we compare the current study to several published data sets in order to assess nutritional status in Maasai populations through time. Comparing Maasai in 2000 to Maasai in 1989 in weight- and height-for-age in children (Age 0–5), there is slight variation in the means but they follow similar trends and fall for the most part below the 5th percentile for the national reference standards (Fig. 2 a–d). One exception lies in Kajiado girls’ mean height-for-age which at age one is above the 5th percentile for Kajiado Maasai females and is above the 50th percentile for the Tanzanian Maasai females. Also female height-for-age increases slightly above the 5th percentile for ages three and four in our sample when compared to the McCabe *et al*. (1989, 1992) Tanzanian sample. At least in the sense of child weight- and height-for-age, nutritional status remains poor, and therefore supports our null hypothesis that nutritional status for this age group is poor and has remained relatively stable through time.

**Fig. 3:**
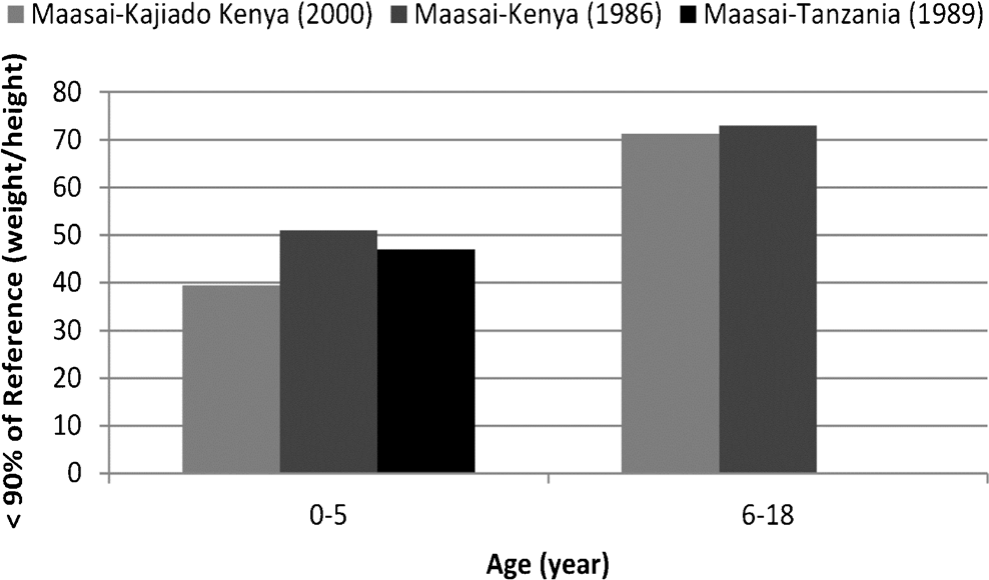
Proportion of individuals below 90 % of reference population in weight-for-height, an indicator of short term nutritional status. Our sample is compared to Nestel’s (1986) study of Maasai in Kenya and McCabe *et al*.’s (1989, 1992) study of a Maasai group in Tanzania. (Nestel’s (1986) anthropometry data were collected every two months over the course of a year and then averaged to get an annual mean. McCabe’s data were collected in June and July 1989 and our data were collected in May and June 2000. We did not account for the potential effects of seasonality or of summed data in the analysis.)

Second, we looked at the proportion of the sub-adult sample that fell below 90 % of the reference population with regard to weight-for-height, an indicator of short term nutritional status (Fig. 3) across the 1985 and 2000 time points (Nestel 1986; McCabe *et al*. 1989, 1992; our study). The data were collected during different times of the year too. Nevertheless, on average, our study had the lowest percentage of individuals who were below this reference standard for both age categories, however all three samples are similar especially for the two samples of Kenyan children from 6 to 18 years of age (there are no data for McCabe *et al*. 1989, 1992). Both of the Kenyan samples exhibit a uniform trend, where there is a decrease in weight-for-height as age increases. This indicates that young children are fed better than juveniles and adolescents, and replicates results of a previous study on Turkana pastoralists carried out by Galvin (1992).

Third, we compared three studies that capture Maasai nutritional status at separate times; the 1930s (Orr and Gilks 1931), 1980s (McCabe *et al*. 1989, 1992), and 2000 (present study). Published reports for Kenyan and Tanzanian Maasai weights, heights, and BMIs for adult male and female participants are compared across the three studies (Table 9). Although statistical comparisons are not possible, there is minimal variation between the samples. This suggests indirectly that despite significant changes in the social-ecological system over time, little change has occurred in regard to nutritional status.

**Table 9:** Descriptive statistics for adult Maasai weight, height, and BMI from sample populations spanning 1930–2000. Data adapted from Homewood (1992), which includes anthropometric data from Orr and Gilks (1931) and McCabe *et al*. (1989) compared to this study

	Sex	Weight			Height			BMI	*n*	SD
		(kg)	*n*	SD	(cm)	*n*	SD			
Kenya 1931^a^	♂	60.2	362		169.3	362		21	362	
	♀	54.2	333		155.5	333		22.4	333	
Tanzania 1989^b^	♂	57.6	88	7.89	171.2	88	6.54	19.7	88	2.3
	♀	49.1	180	6.6	159.9	180	5.5	19.1	180	2.3
Kenya 2000	♂	56.5	96	9.19	172.55	96	5.97	19.02	96	3.06
	♀	47.5	130	8.69	160.26	130	5.54	18.58	130	2.83

Adapted from Homewood (1992)

^a^Data from Orr and Gilks (1931)

^b^Data from McCabe *et al*. (1989, 1992)

#### Diet Recall

**Fig. 4:**
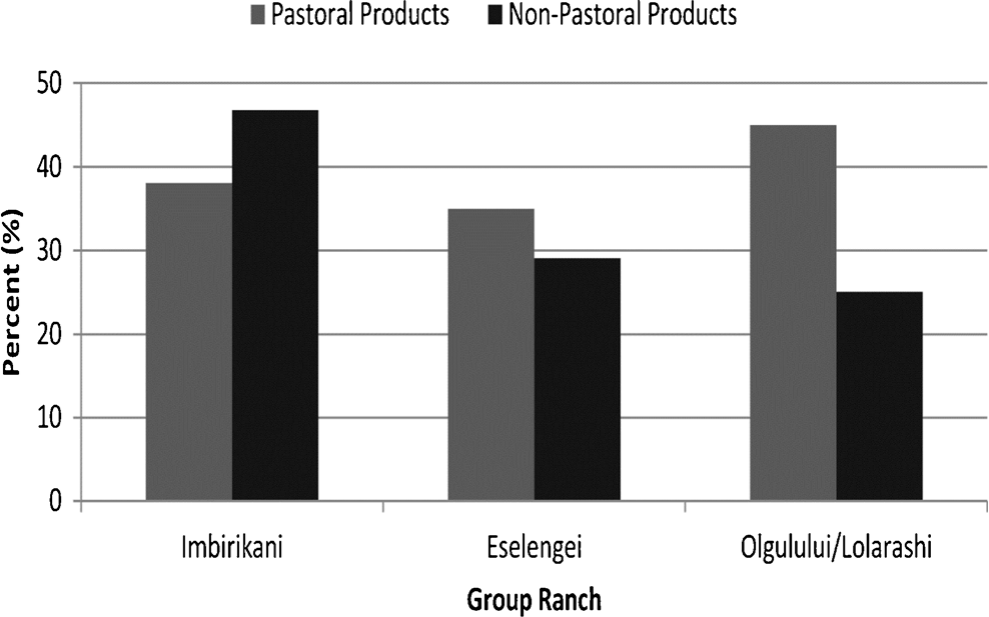
Average reported use of pastoral vs. non-pastoral products by group ranch. Because the diet recall was framed by types and not total foods consumed we do not have a measure of total intake. Therefore we cannot assume that together pastoral foods and nonpastoral equal 100 % of intake. Although cow milk remains the main staple in most households, members in Imbirikani reported using purchased foods on average more than those in other groups (Pastoral foods include milk and meat while non-pastoral foods included tea leaves, sugar, salt, maize meal, fat, rice, beans, potatoes, tomatoes, onions, Sukuma, wheat flour, chicken and combinations thereof.)

Our study was unable to address specific questions regarding the proportion of foods to overall intake and dietary diversity. However, we can glean from the diet recall data an interesting trend wherein households in Imbirikani GR on average rely more heavily on non-pastoral products when compared to the other two GRs. These products are predominantly purchased from markets in either Imbirikani town or weekly regional markets in Namelok and Kimana, and it is likely that they are less protein dense than a traditional pastoral diet that relies heavily on milk, milk fat and meat (Fig. 4). Purchased products, including grains and others, are much more carbohydrate rich. Coincidently, the sample in Imbirikani was closer, on average, to nearby towns (see Fig. 1), has the lowest number of livestock holdings on average, and has a higher proportion of individuals who are sedentary than the other three group ranches (Table 2).

## Discussion

Nutritional status is poor for all age/sex groups. We hypothesized that nutritional status would be poor for the population. All z-scores for nutritional measures were below 1 standard deviation from the mean (except for infant WHZ/BMIZ) and some measures were close to 2 SD below the mean. This was especially prevalent in all male measures of ZUMA and for male and female juvenile WHZ/BMIZ scores as well as those for adolescent boys. Juvenile and adolescent males are the main herders and away from the household during the day, expending energy herding and not available at the household when food is available. A majority of the males from age 2 to adults were malnourished and this was especially prevalent among adolescent boys. Despite socioeconomic and environmental changes, (Table 1) there has not been an improvement in nutritional status from earlier assessments. .

Nutritional status among group ranches is similar. Nutritional status of adults by group ranch did not show significant trends despite differences in any number of group ranch characteristics. All z-scores were below the norm. There were no differences among male and female z-scores except females (BMIZ) and males (ZUMA) in Imbirikani. Imbirikani has informally subdivided agricultural areas and in the southern part of the GR it is very agropastoral. This may account for why adults in Imbirikani tended to have less negative z-scores than adults in other group ranches.

Almost all individuals were underweight or malnourished, with the majority of men in Eselengei and Olugulului/Lolorashi GRs malnourished though over 40 % of sampled men in Imbirikani also had this status. Increasing sedentarization in some areas leads to a decrease in ecological connectivity, and a decrease in the scale of resource use and livestock dependency (Galvin *et al*. 2008; Hobbs *et al*. 2008). However, it is the case that household social networks allow individuals to move their livestock across the landscape, thereby keeping livestock condition high. It may be the case that people are still mobile enough when needed. The observed result is a lack of differences in nutritional status among group ranches although the fewest undernourished occurred in Imbirikani GR whose inhabitants had greatest access to agricultural products and extensive pastoralism.

Hypothesis 3 suggests that nutritional status remained stable over time, though at very poor levels. With regards to weight- and height-for-age in children, nutritional status for Kajiado Maasai and Tanzanian Maasai (McCabe *et al*. 1989) are similar and low despite over a decade of social, economic and environmental changes. This is also the case for the percentage of sub-adults below the 90 % reference population in weight-for-height. Though the percentage below the reference norm is about 40 % among Maasai children in 2000, it is closer to 50 % in 1985 and 1989. For juveniles and adolescents, the proportion below 90 % of the reference is almost identical at about 70 %. Comparing mean weights, heights and BMI for adults over three time periods, 1931, 1989 and 2000, there appear to be few changes. The trends are interesting though with Maasai adults generally weighing more and having higher measures of BMI in 1931, relative to later years. However, heights tended to increase through time. Though we were unable to statistically test this hypothesis because we were dealing with aggregate data, the results still show remarkable consistency through time.

Pastoral diets vary between group ranches (hypothesis 4). Imbirikani individuals generally had more non-pastoral products in their diets than in other group ranches while the Oluglului/Lolarashi sample showed diets highest in pastoral products. This outcome could be attributed entirely to the location of the anthropometric sample. The Imbirikani sample appears to be closest to local towns. However, group ranch characteristics (Table 1) tend to substantiate this result. Imbirikani has the highest infrastructure access (roads, towns), is most sedentary, has the lowest TLU per person on average, the highest agricultural income and least percent of mobile households (although those who did move, moved more often and traveled longer distances in the dry season). It is also the group ranch that has significantly higher BMI and UMA when compared to Eselengei. That difference is not seen in Olgulului/Lorashi, but overall BMI and UMA is higher in Imbirkani than other group ranches hinting at a nutrition transition.

## Conclusions

We analyzed human nutritional data and diets within a context of change to address questions of persistence and a predicted transition of east African pastoral systems. There have been numerous changes to the social-ecological system in Kajiado in the past thirty years. Yet, human health as measured by nutritional status remains stable and poor. What does this imply for persistence or transformation of the pastoral system? It seems that the Maasai have been able to persist and adapt—their nutritional status is approximately the same as 30 years ago. Thus, Maasai do seem to be resilient by being able to absorb change and shocks. Maasai nutritional continuity seems to be operating here. But if improving and building adaptive capacity is considered, then this is *not* occurring, at least at the level of human health.

There has been a theoretical debate about whether ‘small but healthy’ confers functional impairment on the individual with much early literature suggesting that individuals ‘adapt’ to lower energy and protein intake at no functional cost (Seckler 1982; Sukhatme and Margen 1982). Later work of Messer (1986, 1989) and others (*e.g*., Martorell 1989; Vercellotti *et al*. 2014) refute this claim and show overwhelmingly that growth retardation is a major sign of poor health and is associated with compromised immune competence, poor psychological performance, diminished productivity and increased risk of mortality. We agree with the latter group and suggest that while Maasai have adapted, human health remains poor.

Further, pastoralists have tended to be taller than their agricultural neighbors (*e.g*., Galvin 1992; Hadley and Crooks 2012; Little and Johnson 1987) showing the benefits of milk-consumption to linear growth. However, milk consumption may not offset highly prevalent food insecurity and exposure to illness. Lawson *et al*. (2014) compared health and nutritional status among Maasai and other ethnic groups in Tanzania and showed that over half of the Maasai children were stunted compared to about 20% of agricultural children. Maasai children had much less access to carbohydrates, were at higher risk of certain illnesses such as pneumonia and diarrhea, and were very food insecure. They also inhabited the dry regions of Tanzania relative to the other ethnic groups. Our sample population may have similar characteristics.

Many human populations are initially buffered against the degradation of ecosystem services and there is a temporal lag between ecosystem change and the resultant impacts on humans (Myers and Patz 2009). It is difficult to show causation or even correlation between human and ecosystem health due to social, economic, political and cultural activities that buffer vulnerability to the collapse of ecosystem services. In Kajiado, the case may be that human biological change is lagging behind the socio-economic and environmental changes that are cascading through the system. It may be that Maasai nutritional state will improve in the future but it may remain vulnerable to surprise and crisis in the short term (Herrfahrdt-Pähle and Pahl-Wostl 2012).

Imbirikani group ranch has better access to infrastructure and markets and may be undergoing a dietary transition. This implies several other changes that we would expect to occur (i.e., livestock holdings decreases, sedentism increases, participation in agriculture increases). And in fact these changes are occurring. People who live closer to larger towns, markets and main roads are no longer able to live directly from their livestock and thus must sell livestock products, conduct some agriculture and purchase food to supplement their household food supply more often than those pastoralists living further away However, people are choosing to live near towns for a variety of reasons including access to schools and health care (Fratkin *et al*. 1999; Little *et al*. 2001). The result is that ecosystem services (water, forage, nutrition directly from livestock) needed for livestock production are not readily available. Yet, nutritional status, remains poor, is similar across group ranches and across age/sex groups and is similar to other pastoral nutritional studies (*e.g*., Galvin 1992; Galvin and Little 1999; Galvin *et al*. 2002; Knapp *et al*. 2015).

We lack data on demographic shifts and changes in epidemiological patterns, two population processes that affect and are affected by nutritional change. The focus on these shifts conceal the underlying processes of change, that is, social and economic integration in market economies as drivers of increased rates of overweight and obesity, associated with reductions in physical activity. Social and economic activities are also associated with increased dependence on purchased food. Our information also suggested, environmental changes, in addition to social and economic transformations are part of the suite of processes that affect nutritional change, especially for populations who are increasingly integrated into market economies in their local places. This paper references numerous changes in the ecological productivity and access as well and social and economic changes. Diets are changing yet there is food insecurity. Market integration is not correlated with changing nutritional status. It is likely that other measures of a transition may need to be taken into account like education, health care, infrastructure (i.e., roads and water) or that this is an early stage of a transition. However, the fact that nutritional status is similar across group ranches also suggests that Maasai values of obligatory sharing and reciprocity in food, pasture, and animals seem to still be in place. We see a pastoral system that is transforming and yet illustrates continuity under change.
